# Long-term distress throughout one’s life: health-related quality of life, economic and caregiver burden of patients with neurofibromatosis type 1 in China

**DOI:** 10.3389/fpubh.2024.1398803

**Published:** 2024-08-21

**Authors:** Wanxian Liang, Shihuan Cao, Yusi Suo, Lining Zhang, Lujia Yang, Ping Wang, Hanfei Wang, Han Wang, Guannan Bai, Qingnan Li, Jiayin Zheng, Xuejing Jin

**Affiliations:** ^1^Centre for Evidence-Based Chinese Medicine, School of Traditional Chinese Medicine, Beijing University of Chinese Medicine, Beijing, China; ^2^International Institute of Evidence-Based Traditional Chinese Medicine, School of Traditional Chinese Medicine, Beijing University of Chinese Medicine, Beijing, China; ^3^Children’s Hospital, Zhejiang University School of Medicine, National Children’s Regional Medical Center, National Clinical Research Center for Child Health, Hangzhou, Zhejiang, China; ^4^China Alliance for Rare Diseases, Beijing, China; ^5^Beijing Society of Rare Disease Clinical Care and Accessibility, Beijing, China

**Keywords:** neurofibromatosis type 1, caregiver burden, economic burden, EQ-5D, health-related quality of life

## Abstract

**Introduction:**

Neurofibromatosis type 1 (NF1) is a rare genetic disorder, with lack of evidence of disease burden in China. We aimed to describe the economic burden, health-related quality of life (HRQL), and caregiver burden of NF1 patients in China.

**Methods:**

We conducted an online cross-sectional survey employing the China Cloud Platform for Rare Diseases, with 223 caregivers of NF1 pediatric patients (patients under 18), and 226 adult patients. Economic burden was estimated using direct and indirect costs related to NF1 in 2021, and the Work Productivity and Activity Impairment Questionnaire: General Health V2.0 (WPAI-GH). HRQL measures included EQ-5D-Y proxy version and PedsQL^™^ 4.0 Generic Core Scales (PedsQL GCS) proxy version for pediatric patients, and EQ-5D-5L and PedsQL^™^ 3.0 Neurofibromatosis Module (PedsQL NFM) for adult patients. Caregiver burden was estimated by Zarit Burden Interview (ZBI).

**Results:**

For pediatric patients, the average direct cost in 2021 was CNY 33,614 (USD 4,879), and employed caregivers’ annual productivity loss was 81 days. EQ-5D-Y utility was 0.880 ± 0.13 and VAS score was 75.38 ± 20.67, with 52.6% patients reporting having problems in “pain/discomfort” and 42.9% in “anxiety/depression.” PedsQL GCS total score was 68.47 ± 19.42. ZBI score demonstrated that 39.5% of caregivers had moderate-to-severe or severe burden. For adult patients, average direct cost in 2021 was CNY 24,531 (USD 3,560). Patients in employment reported an absenteeism of 8.5% and presenteeism of 21.6% according to the results of WPAI-GH. EQ-5D-5L utility was 0.843 ± 0.17 and VAS score was 72.32 ± 23.49, with more than half of patients reporting having problems in “pain/discomfort” and “anxiety/depression” dimensions. PedsQL NFM total score was 68.40 ± 15.57.

**Conclusion:**

Both pediatric and adult NF1 patients in China had a wide-ranging economic burden and low HRQL, especially in the psychological dimension. Caregivers for NF1 pediatric patients experienced considerable caregiver burden. More attention and support from policymakers and stakeholders are required to relieve NF1 patients’ and caregivers’ distress.

## 1 Introduction

Around 260–450 million people suffer from rare diseases worldwide (1). Neurofibromatosis Type 1 (NF1), an autosomal dominant genetic disorder with a global prevalence of about 1 in every 3,000 newborns (2–4), meets the definition of rare diseases in China, the US, and the European Union (5–8). NF1 is caused by an error on chromosome 17, which affects the development of multiple systems including skin, bone, eyes, nerves, and cardiovascular system (9–11).

Clinical manifestations of NF1 vary widely, ranging from mild cutaneous abnormalities like café-au-lait skin spots to severe symptoms like benign or even malignant tumors within the peripheral or central nervous system (11). Other manifestations of NF1 include headaches, learning difficulties, speech articulation difficulties, impaired executive function and attention, skeletal features, and other less common symptoms. The life expectancy of individuals with NF1 is 8–15 years shorter than the general population (4, 12, 13). NF1 not only affects physical health but also causes a significant psychological and economic burden on individuals (14). Roughly 19–55% of NF1 patients reported experiencing depression, while 15% reported anxiety at clinical level (15–17). Furthermore, NF1 impacts on adult patients’ incomes (18) and places economic and caregiver burden on NF1 pediatric patients’ families (9, 19).

The disease burden among patients with NF1 in China is not well understood. Only one Chinese study reported the health-related quality of life (HRQL) of 27 NF1 patients (age 3–49 years), indicating that NF1 patients have impaired HRQL (20). As innovative therapies for NF1 gradually emerged recently, there is boosting demand for comprehensive evidence of the burden of NF1 in China, which can support reimbursement decision-making of those therapies and the development of clinical practice guidelines.

Since there is a lack of solid evidence to describe the landscape of the disease burden for NF1 patients in China, the present study aimed to measure economic and humanistic burden, including HRQL and caregiver burden of NF1 patients in China.

## 2 Materials and methods

### 2.1 Study overview

A cross-sectional survey was conducted online among adult patients with NF1 and caregivers of NF1 pediatric patients (patients under 18 years) from November 2022 to January 2023. Participants were recruited through Neurofibromatosis Shenzhen Care Center (NSCC), a patient network with members across the country.

This study was conducted according to the Declaration of Helsinki and approved by the Ethics Committee of Beijing University of Chinese Medicine (2022BZYLL1005). All eligible participants volunteered to participate in the study and provided electronically signed consent forms before completing the questionnaire.

### 2.2 Sample size calculation

Two factors were considered when deciding the study’s sample size. Firstly, the minimum sample size was calculated using the sample size calculation equation for cross-sectional study (21, 22), $n=\frac{Z_{1-\alpha /2}^{2}*S^{2}}{d^{2}}$ , with *S* as the standard deviation of EQ-5D-5L utility score in NF1 patients, 0.24, and *d* as the minimal important difference of EQ-5D-5L utility score in Chinese population, 0.058 (23, 24). We set the significance level as 0.05 so $Z_{1-\alpha /2}$ was 1.96, n was calculated as 66. We assumed a 20% non-response rate and the ratio of adults and children was 1:1 according to suggestions from clinicians and the NSCC. The minimum of the sample size was calculated as 165. Except for the EQ-5D, the questionnaire also collected information about economic burden and caregiver burden and included more than one HRQL measure. Therefore, the sample size of the study should be larger than the minimum.

Secondly, participants of the study were recruited through the nationwide patient network, NSCC, with more than 15, 000 registered patients from all over China and around 1,000 active members (the proportion of pediatric patients and adult patients are around 50 and 50%, respectively). The study sample of other cross-sectional surveys conducted by NSCC previously was around 500, which was taken as the maximum of the sample size. Considering the number of patients who volunteered to participate in the study would be less than the active members, we finally decided that the target sample size was 450 patients.

### 2.3 Participants

For diagnosed NF1 pediatric patients, caregivers were recruited as proxy in this study. The inclusion criteria for the caregivers were: (1) primary caregivers who were familiar with the health condition of the patients; (2) able to understand the questionnaire; and (3) volunteered to participate in the study and signed the informed consent.

For diagnosed NF1 adult patients, inclusion criteria were: (1) able to understand the questionnaire; and (2) volunteered to participate in the study and signed the informed consent.

Any participants who were unable to complete the survey for any reason were excluded from the study.

### 2.4 Questionnaire

The caregiver-reported questionnaire consisted of four parts: (1) demographic information of patients and caregivers including age, gender, disease duration, residence, household income, schooling status, and health insurance; (2) economic burden of NF1 patients; (3) proxy-reported HRQL of NF1 patients (EQ-5D-Y and PedsQL™ 4.0 Generic Core Scales); and (4) caregiver’s burden (Zarit Burden Interview, ZBI).

The adult patient-reported questionnaire consisted of (1) demographic information; (2) economic burden as well as Work Productivity and Activity Impairment Questionnaire: General Health V2.0 (WPAI-GH V2.0); and (3) self-reported HRQL of NF1 patients [EQ-5D-5L and PedsQL^™^ 3.0 Neurofibromatosis Module (adult version)].

HRQL measures used in this study should meet the following conditions: (1) have an existing Chinese version provided by the copyright holders; (2) have been used in previous studies with neurofibromatosis patients. All the Chinese-version HRQL measures in this study were obtained from official sources. The EQ-5D-Y and EQ-5D-5L were obtained from the EuroQol Research Foundation.^1^ The PedsQL^™^ 4.0 Generic Core Scales, PedsQL^™^ 3.0 Neurofibromatosis Module (adult version), and ZBI were obtained from the Mapi Research Trust.^2^ The WPAI-GH V2.0 was obtained from the website of Reilly Associates.^3^ The caregiver-reported questionnaire and the adult-patient-reported questionnaire were both in Chinese.

#### 2.4.1 Economic burden

The NF1-related direct costs for 2021 were collected, including inpatient cost (number of hospital admissions, length of each inpatient stay, and average expenses for each hospitalization), outpatient cost (number of outpatient visits, average expenses for each outpatient visit), formal care cost, other direct medical costs (pharmacy expenses, and medical equipment expenses), and direct nonmedical costs related to NF1 (traffic and accommodation expenses, fees of rehabilitation courses for children with deficiencies in skeletal or intellectual development, etc.).

Regarding the indirect cost, for adult patients and the primary caregivers for pediatric patients, we collected the work-loss days in 2021 and estimated the productivity loss using the human capital approach based on the average salary of 2021 in non-private sectors or private sectors (25, 26). Specifically, we converted work-loss days into currency by dividing the average annual salary by 365 days and then multiplying it by the number of work-loss days. The average salary of 2021 in non-private sectors or private sectors in China was CNY 106,837 (USD 15,506) and CNY 62,884 (USD 9,127), respectively (26). For pediatric patients, school-loss days in 2021 were also collected. NF1-related work-loss days and school-loss days for patients include absences directly resulting from the NF1 condition itself, medical consultations, follow-up examinations related to NF1, and other absences specifically linked to managing NF1. Work-loss days of primary caregivers for pediatric patients were caused by caring for NF1 patients. Besides, WPAI-GH V2.0 was used to measure the impairment of productivity and activity of NF1 adult patients related to their overall health condition.

#### 2.4.2 Measures

##### 2.4.2.1 WPAI-GH V2.0

The WPAI-GH V2.0 is a six-item instrument designed to measure work time missed and productivity and activity impairment in the past 7 days for the general population. WPAI-GH is scored on four metrics: percentage of work time missed due to one’s health condition, i.e., absenteeism; percentage of reduction in work effectiveness due to one’s health condition, i.e., presenteeism; percentage of work productivity loss due to one’s health condition, i.e., overall work impairment/absenteeism plus presenteeism; and proportion of impairment in activities of daily living due to one’s health condition, i.e., activity impairment (27). Absenteeism, presenteeism, work productivity loss, and activity impairment that obtained from the results of WPAI-GH were relevant to one’s overall health condition, not just associated with NF1 (28).

##### 2.4.2.2 EQ-5D instruments

The EQ-5D instrument is a series of generic indirect preference-based HRQL measures that can provide utility scores which can be used to calculate quality-adjusted life year (QALY) in economic evaluations (29, 30). It measures HRQL in five dimensions: “mobility,” “self-care,” “usual activities,” “pain/discomfort,” and “anxiety/depression” (31). The EQ-5D-Y is the child-friendly version of EQ-5D, using different expressions in “self-care” (“looking after him/herself”), “usual activities” (“doing usual activities”), “pain/discomfort” (“having pain or discomfort”), and “anxiety/depression” (“feeling worried, sad or unhappy”) dimensions. It has three response levels of severity to each dimension (32). This study used a proxy version of EQ-5D-Y to measure the health status of patients aged between 4 and 18. The EQ-5D-5L is an adult version with five response levels of severity to each dimension (31).

Both the EQ-5D-Y and EQ-5D-5L value sets for the Chinese population had been published so that utility scores could be calculated (33–35). Utility scores usually range on a 0–1 scale, where 1 for full health and 0 for death; sometimes we can see negative utility scores, meaning health states worse than death. EQ-5D also has a visual analog scale (VAS) ranging from 0 (worst imaginable health) to 100 (best imaginable health) to record patient today’s self-rated health (31, 32). EQ-5D-Y and EQ-5D-5L had good reliability and validity measuring HRQL of Chinese populations with different health conditions (36–41).

##### 2.4.2.3 PedsQL^™^ 4.0 Generic Core Scales

The proxy version of the Pediatric Quality of Life Inventory Generic Core Scales (PedsQL^™^ 4.0 Generic Core Scales, PedsQL GCS) was applied to estimate HRQL of NF1 patients aged from 2 to 18. The proxy version of PedsQL GCS showed good reliability in children and adolescents with different health conditions (42–45). PedsQL GCS was not only a validated HRQL measure for healthy children but also one of the most recommended HRQL measures to be used among children with NF1 at different ages (46, 47). It is a 23-item instrument containing 4 scales: physical(8 items), emotional (5 items), social (5 items), and school functioning (5 items), which can be further divided into 2 domains: physical health and psychosocial health (42, 48).

The 5-point Likert-type response scales (ranging from 0 to 4, 0 = almost never a problem, 1 = seldom a problem, 2 = sometimes a problem, 3 = often a problem, 4 = almost always a problem) were provided as choices for each item in PedsQL GCS (49). All the items need to be linearly transformed into a 0–100 scale (0 = 100, 1 = 75, 2 = 50, 3 = 25, 4 = 0) (48). Total scores and scale scores are calculated as the sum of the item scores divided by the number of items answered, a higher score indicates a better health state (48).

##### 2.4.2.4 PedsQL^™^ 3.0 Neurofibromatosis Module

The self-reported adult version of the Pediatric Quality of Life Inventory Neurofibromatosis Module (PedsQL^™^ 3.0 Neurofibromatosis Module, PedsQL NFM) was used for NF1 adult patients in this study. PedsQL NFM is a disease-specific HRQL measure to be used among individuals with NF1 aged above 5 years old, with good reliability and validity (45, 46). The PedsQL NFM was recommended by the Response Evaluation in Neurofibromatosis and Schwannomatosis (REiNS) International Collaboration as the highest-rated NF1-specific measure specifically for patients with NF1 in clinical trials (46). PedsQL NFM contains 18 dimensions and 104 items in total (45).

PedsQL NFM has the same scoring system as PedsQL GCS. Total scores and dimension scores are calculated as the sum of the item scores divided by the number of items answered, a higher score indicates a better health state (48).

##### 2.4.2.5 Zarit Burden Interview

ZBI is a 22-item instrument widely used to measure the caregiver’s subjective burden, that is, the assessment a caregiver forms about how caring for others affects their personal lives in aspects of burden in the relationship, emotional well-being, social and family life, finances, loss of control over one’s life (50, 51). The Chinese version shows good reliability and validity (52, 53). It measures the caregiver’s burden in five domains with a total score range from 0 to 88 (0–4 points for each item) (50). A higher total score indicates a heavier caregiver burden. Caregiver burden can be divided into 4 levels according to the ZBI total score: little or no (0 to 20), mild to moderate (21–40), moderate to severe (41–60), and severe (61–88) (54).

### 2.5 Data collection

The China Cloud Platform for Rare Diseases, a one-on-one online interview system developed by the China Alliance for Rare Diseases (CHARD), was employed in data collection. The online interview system allowed interviewers to see participants’ online responses in real time at their end. The online survey was mainly self-answered by the participants. The participants maintained a voice connection with an interviewer while answering the online questionnaire and could get help from the interviewer at any time. Six interviewers (master and PhD students from Beijing University of Chinese Medicine) were trained, and six volunteers from NSCC helped recruit participants.

### 2.6 Statistical analysis

Descriptive statistics were used to demonstrate the economic and human burden of NF1 patients. Where hypothesis testing is required, for continuous variables (e.g., EQ-5D-Y utility scores and ZBI total scores), the t-test or the analysis of variance were used for normal distribution, and the Wilcoxon rank-sum test or the Kruskal-Wallis test were used for non-normal distribution. Averages of the scores of the PedsQL GCS were compared with those scores of healthy controls that were extracted from the published literature (55). All statistical tests were two-sided and *p* < 0.05 was used to indicate the significance, conducted using Stata^®^ 17.0 SE.

## 3 Results

### 3.1 Sample characteristics

A total of 449 participants were included in the study, among which 223 were caregivers of pediatric patients and 226 were adult patients. The study population was drawn from 29 provinces across China, making it a relatively geographically representative sample.

For the 223 pediatric patients, the mean age was 6.26 ± 4.25 and 54.3% of them were females. Nearly half of the pediatric patients (50.7%, 113/223) were in school while 47.1% (105/223) were preschoolers and 2.2% (5/223) were suspended from school (Table 1). About 14.8% (33/223) of the pediatric patients had one or more family members diagnosed with NF1 (Table 2). A diagnosis of NF1 was given to 34.1% of the patients (76/233), in the dermatological department, which made up the largest proportion of all. The percentage of pediatric patients who reported misdiagnosis before a confirmed diagnosis was 17.0% (38/223). Mothers filled out 80.3% (179/223) of the questionnaires and 70.4% (157/223) of caregivers for NF1 pediatric patients were employed (Table 3).

**Table 1:** Demographic information for patients with NF1.

	NF1 pediatric patients	NF1 adult patients
	*n* = 223	*n* = 226
Age, mean ± SD, years	6.26 ± 4.25	31.54 ± 7.60
Female, *n* (%)	121 (54.26%)	144 (63.72%)
BSA, mean ± SD, m^2^	0.87 ± 0.34	1.57 ± 0.19
Height, mean ± SD, cm	115.88 ± 27.53	159.91 ± 9.42
Weight, mean ± SD, kg	23.95 ± 14.19	55.84 ± 11.35
Diagnosis age	3.60 ± 3.47	20.19 ± 8.55
Family annual income (CNY), *n* (%)		
<10,000	10 (4.50%)	38 (16.81%)
[10,000–30,000)	21 (9.40%)	37 (16.37%)
[30,000–50,000)	43 (19.30%)	52 (23.01%)
[50,000–100,000)	64 (28.70%)	59 (26.11%)
[100,000–200,000)	49 (22.00%)	26 (11.50%)
[200,000–300,000)	23 (10.30%)	7 (3.10%)
> = 300,000	13 (5.80%)	7 (3.10%)
Residence, *n* (%)		
Rural	59 (26.46%)	91 (40.27%)
Urban	164 (73.54%)	135 (59.73%)
Marriage, *n* (%)		
Married	NA	111 (49.12%)
Others^#^	NA	115 (50.88%)
Education, *n* (%)		
Primary school or below	199 (89.24%)	7 (3.10%)
Junior high school	21 (9.42%)	62 (27.43%)
Senior high school/technical secondary school	3 (1.35%)	48 (21.24%)
University/college and above^*^	NA	109 (48.23%)
Schooling status, *n* (%)		
Suspended	5 (2.24%)	NA
In school	113 (50.67%)	NA
Preschooler	105 (47.09%)	NA
Working status, *n* (%)		
Not employed	NA	56 (24.78%)
Employed part-time	NA	29 (12.83%)
Employed full-time	NA	104 (46.02%)
Others^##^	NA	37 (16.37%)
Covered by basic medical insurance, *n* (%)		
Yes	215 (96.41%)	221 (97.79%)
Covered by supplementary medical insurance, *n* (%)		
Yes	111 (49.78%)	75 (33.19%)

NA, not applicable; BMI, body mass index; BSA, body surface area; (Mosteller’s formula [weight (kg) × height (cm)/3,600]^1/2^ was used to calculate BSA), NF1, neurofibromatosis type 1.

* University/college and above included patients who received higher education.

# Others of marriage includes divorced or separated, widowed, and single.

## Others of working status includes homemaker, retired, student, loss of labor, and other working status.

**Table 2:** NF1-related medical history of patients.

	NF1 pediatric patients	NF1 adult patients
	*n* = 223	*n* = 226
Numbers of immediate family diagnosed with NF1 (included parents, grandparents, and siblings), *n* (%)		
0	190 (85.20%)	103 (45.58%)
1	25 (11.21%)	71 (31.42%)
More than 1	8 (3.59%)	52 (23.00%)
Symptoms for the first visit, *n* (%)		
Skin	189 (84.75%)	169 (74.78%)
Skeletal	44 (19.73%)	26 (11.50%)
Learning or attention deficiency	23 (10.31%)	20 (8.85%)
Visual	22 (9.87%)	14 (6.19%)
Pain	15 (6.73%)	43 (19.03%)
Psychological	3 (1.35%)	30 (13.27%)
Others	28 (12.56%)	30 (13.27%)
Diagnosis department, *n* (%)		
Dermatological department	76 (34.08%)	85 (37.61%)
Neurosurgery department	42 (18.83%)	41 (18.14%)
Orthopedic and plastic surgery department	23 (10.31%)	24 (10.62%)
Others^#^	82 (36.77%)	76 (33.63%)
Have experiences of misdiagnosis, *n* (%)		
Yes	38 (17.04%)	45 (19.91%)
Places where a confirmed diagnosis was made, *n* (%)		
First-tier cities^*^	104 (46.64%)	76 (33.63%)
Others	19 (53.36%)	150 (66.37%)
Have experiences of off-site medical treatment since the onset of illness, *n* (%)		
Yes	142 (63.68%)	136 (60.18%)

NF1, neurofibromatosis type 1.

* First-tier cities include Beijing, Shanghai, Guangzhou and Shenzhen.

# Other diagnosis departments include neurology department, pediatric department, oncology department, orthopedics department and other departments.

**Table 3:** Characteristics of caregivers of NF1 pediatric patients (*n* = 223).

	Caregiver of NF1 pediatric patients
Age, mean ± SD, years	36.66 ± 6.18
Character of caregiver, *n* (%)	
Mother	179 (80.27%)
Father	40 (17.94%)
Others^#^	4 (1.79%)
Caregiver diagnosed with NF1, *n* (%)	
Yes	18 (8.07%)
Marital status, *n* (%)	
Married	203 (91.03%)
Others^##^	20 (8.97%)
Employment, *n* (%)	
Employed full-time	131 (58.74%)
Not employed	32 (14.35%)
Employed part-time	26 (11.66%)
Others^###^	34 (15.25%)

NF1, neurofibromatosis type 1.

# Others include grandparents and siblings.

## Others include divorced or separated, widowed, and single.

### Others include homemaker, retired and other employed status.

As for the 226 adult patients, the mean age was 31.54 ± 7.60 and 63.7% of them were females. The percentage of adult patients employed was 58.8% (133/226) (Table 1). In the adult group, 54.4% (123/226) had one or more family members diagnosed with NF1 (Table 2). A diagnosis of NF1 was given to 37.6% of the patients (85/226), in the dermatological department, which made up the largest proportion of all. About one-fifth of adult patients in the present study (19.9%, 45/226) reported misdiagnosis before they got a confirmed diagnosis.

### 3.2 Economic burden

#### 3.2.1 Direct cost

**Figure 1:**
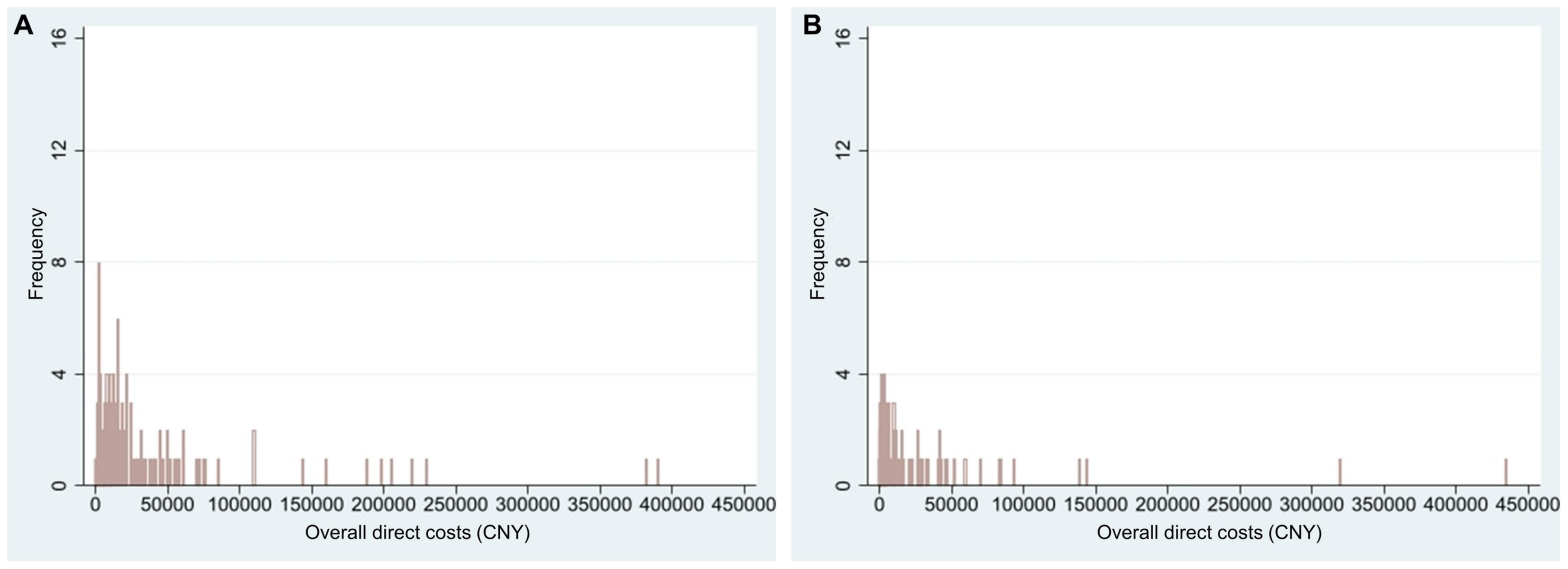
Histogram of NF1-related overall direct costs in 2021 (CNY). Only patients with actual expenses were calculated. **(A)** Overall direct costs of NF1 pediatric patients (*n* = 152, mean = 33,614, range 30 to 390,000); **(B)** Overall direct costs of NF1 adult patients (*n* = 107, mean = 24,531, range 10 to 435,000). NF1, neurofibromatosis type 1.

For NF1 pediatric patients, the average overall direct cost in 2021 was CNY 33,614 (USD 4,879, *n* = 152) (Figure 1). For all pediatric patients in this study, the annual NF1-related average overall direct cost was CNY 22,912 (USD 3,325, *n* = 223). Supplementary Figures S1–S5 displayed the distribution of different types of direct costs in 2021 (calculated in CNY). The average number of admissions was 2.15 ± 2.67 in 2021 and the average length of each inpatient stay was 10.27 ± 8.22 days (*n* = 48).

For NF1 adult patients, the average overall direct cost in 2021 was CNY 24,531 (USD 3,560, *n* = 107) (Figure 1). For all adult patients in this study, the annual NF1-related average overall direct cost was CNY 11,614 (USD 1,686, *n* = 226). Supplementary Figures S1–S5 displayed the distribution of different types of direct costs in 2021 (calculated in CNY). The average number of admissions was 1.62 ± 1.57 in 2021 and the average length of each inpatient stay was 23.48 ± 59.34 days (*n* = 29).

#### 3.2.2 Indirect cost

##### 3.2.2.1 Productivity loss

For caregivers of pediatric patients, 113 interviewees reported productivity loss related to caring for NF1 patients, of which the average number was 81 days (productivity loss of the primary caregiver was calculated) in 2021, CNY 13,822 to 23,709 (USD 2,006–3,441). Fifty pediatric patients were reported to have NF1-related school-loss days in 2021, with an average of 58 days (Table 4).

**Table 4:** Indirect cost of patients with NF1 in 2021 (calculated in days).

	NF1 pediatric patients			NF1 adult patients		
	Mean ± SD	Median (Min, Max)	*Number of people*	Mean ± SD	Median (Min, Max)	*Number of people*
Caregiver work-loss days^*^	81.22 ± 131.90	17 (2, 365)	113	31.11 ± 62.86	15 (1, 365)	37
Self-school-loss days^#^	58.23 ± 89.64	30 (0.5, 365)	50	NA	NA	NA
Self-work-loss days^##^	NA	NA	NA	33.24 ± 62.89	14.5 (1, 365)	34

NF1, neurofibromatosis type 1.

* Caregiver work-loss days mean the work loss of the primary caregiver of NF1 patients.

# Self-school-loss days were calculated only in NF1 pediatric patients.

## Self-work-loss days were calculated only in NF1 adult patients.

For adult patients, a total of 37 interviewees reported caregivers’ productivity loss due to caring for NF1 patients, of which the average number was 31 days (productivity loss of the primary caregiver was calculated) in 2021, CNY 5,341 to 9,074 (USD 775–1,317). Thirty-four adult patients reported patient’s work-loss days in 2021 related to NF1, with an average of 33 days, CNY 5,685–9,659 (USD 825–1,402).

##### 3.2.2.2 WPAI-GH

**Figure 2:**
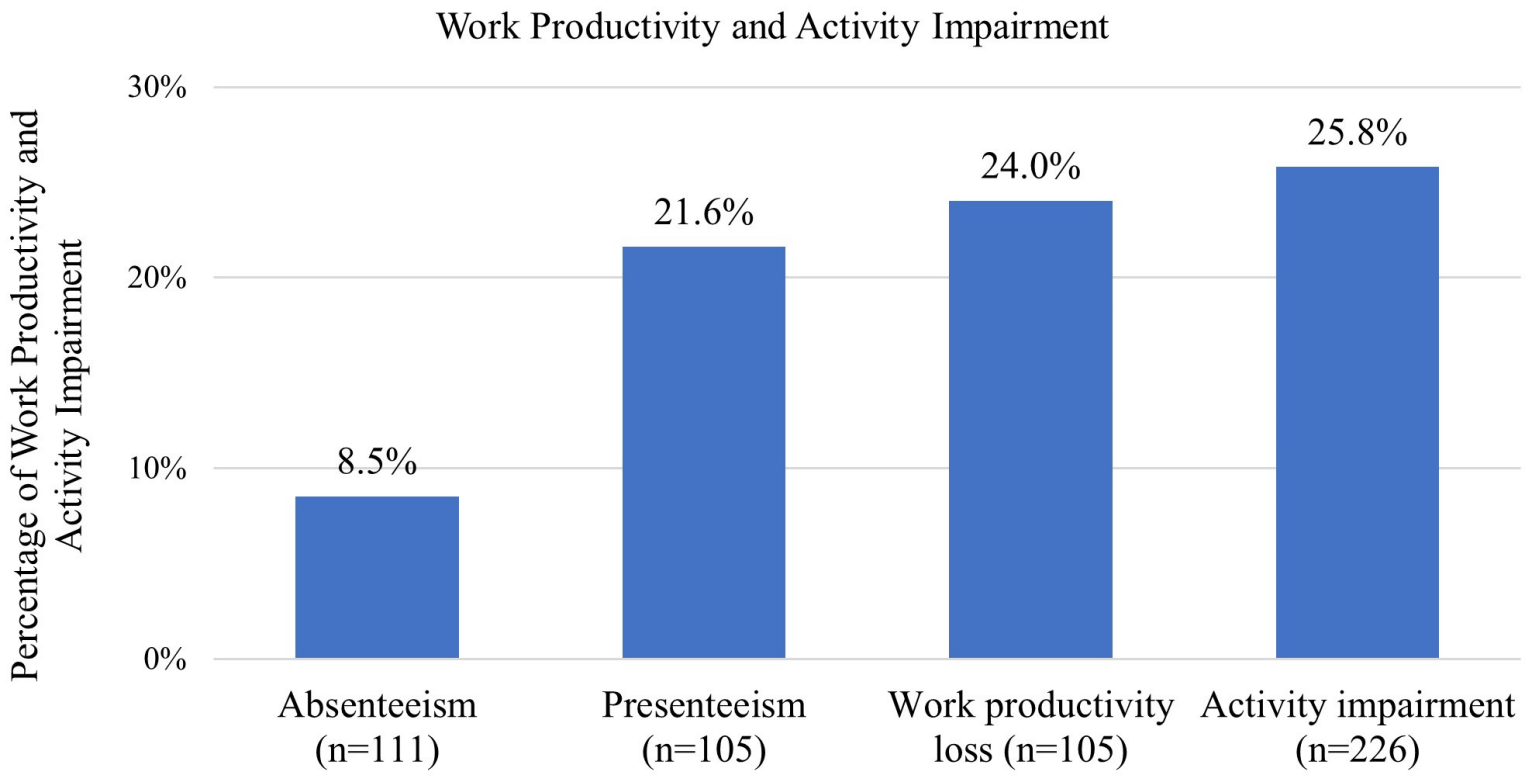
Work productivity and activity impairment for NF1 adult patients based on the WPAI-GH. Absenteeism, presenteeism, and work productivity loss were only assessed among the adult patients who were employed and working in the 7 days prior to answering the questionnaire. Activity impairment was assessed among all adult patients. NF1, neurofibromatosis type 1.

Among adult patients gainfully employed, an average absenteeism of 8.5% (*n* = 111) and an average presenteeism of 21.6% (*n* = 105) were reported, contributing to 24.0% (*n* = 105) work productivity loss in the last 7 days. Among all the adults in the study population (*n* = 226), the average activity impairment was 25.8% (Figure 2). Absenteeism, presenteeism, work productivity loss, and activity impairment were caused by overall health problems.

### 3.3 HRQL

#### 3.3.1 EQ-5D

**Figure 3:**
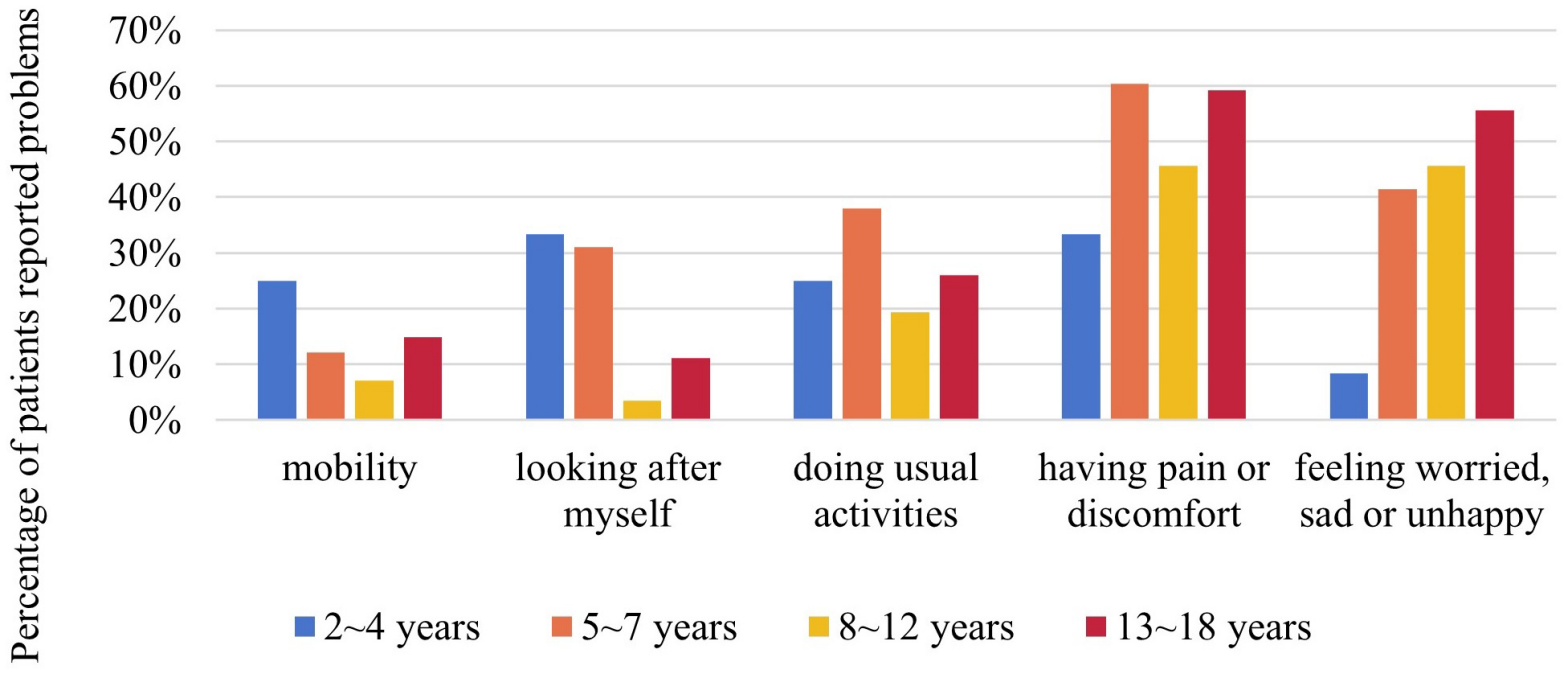
Percentage of NF1 pediatric patients reported problems in different dimensions of EQ-5D-Y by age group. EQ-5D-Y was only for NF1 pediatric patients aged between 4 and 18 (*n* = 154). NF1, neurofibromatosis type 1.

**Figure 4:**
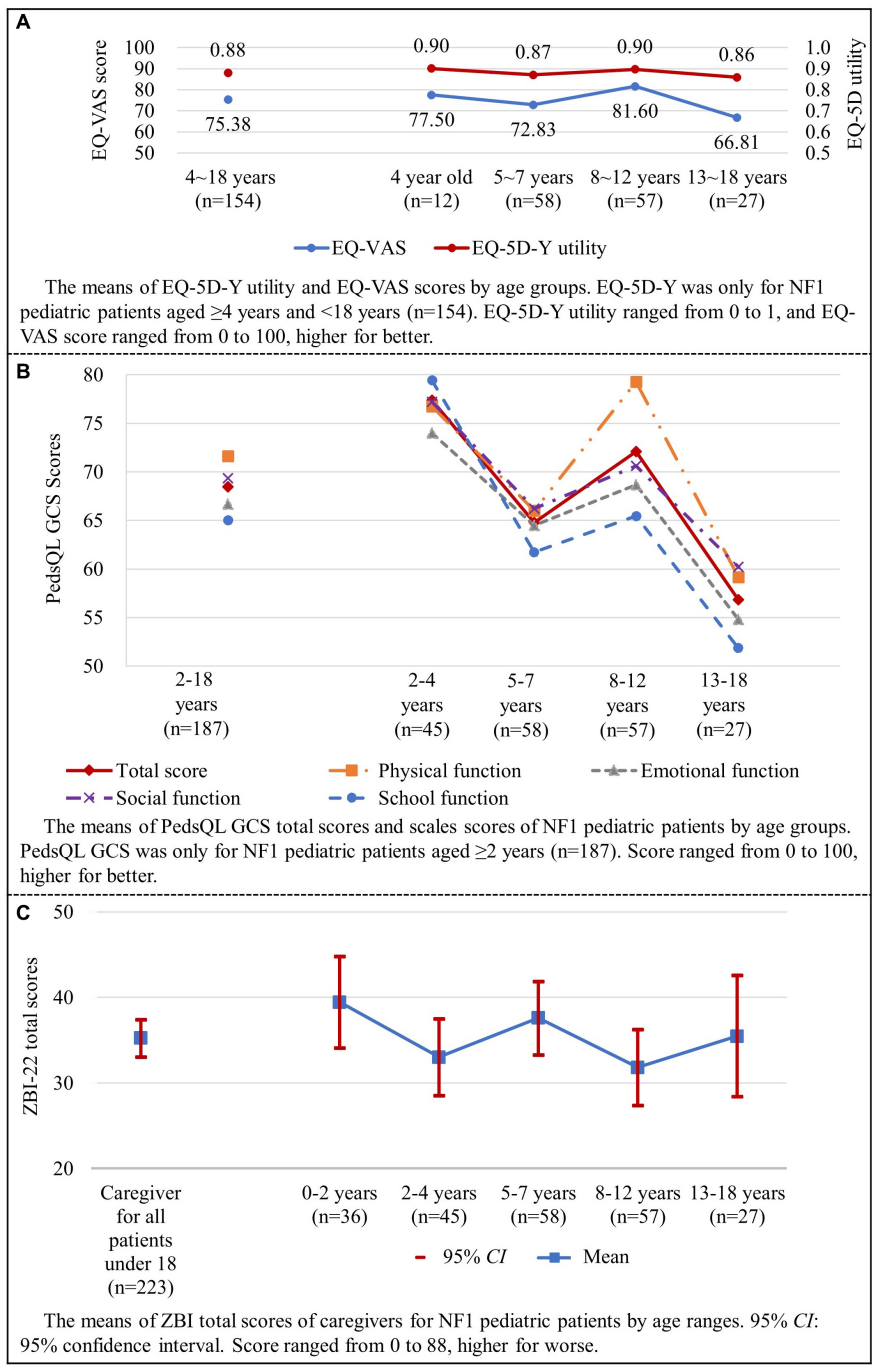
HRQL and caregiver burden of NF1 pediatric patients (shown in **A–C**). HRQL, health-related quality of life; NF1, neurofibromatosis type 1.

The EQ-5D-Y proxy version was administered to 154 pediatric patients no less than 4 years old. The dimension that patients had the most problems with was “having pain or discomfort” (52.6%), followed by “feeling worried, sad or unhappy” (42.9%) (Figure 3). EQ-5D-Y utility and VAS score were 0.880 ± 0.133 (*n* = 154) and 75.38 ± 20.67 (*n* = 154), respectively (Figure 4A).

The EQ-5D-5L was administered to all adult patients (*n* = 226). The percentage of patients who reported having problems with “pain/discomfort” and “anxiety/depression” were 58.9 and 74.3%, respectively. For adult patients, EQ-5D-5L utility and VAS score were 0.843 ± 0.167 and 72.32 ± 23.49, respectively (Table 5).

**Table 5:** Results of EQ-5D-5L utility and VAS score in NF1 adult patients (*n* = 226).

	Male Mean ± SD (*n* = 82)	Female Mean ± SD (*n* = 144)
EQ-5D-5L		
Utility	0.828 ± 0.185	0.851 ± 0.157
VAS	73.40 ± 23.94	71.71 ± 23.28
Dimensions (percentage of patients who reported having problems)		
Mobility	29.27%	11.81%
Self-care	9.76%	5.56%
Usual activities	24.39%	12.50%
Pain/discomfort	54.88%	61.11%
Anxiety/depression	64.63%	79.86%

NF1, neurofibromatosis type 1.

#### 3.3.2 PedsQL GCS scores for pediatric patients

For pediatric patients, the total score of PedsQL GCS was 68.47 ± 19.42. The total scores of PedsQL for different age ranges were as follows: 77.38 ± 19.13 for patients aged 2–4, 64.79 ± 18.22 for patients aged 5–7, 72.08 ± 14.26 for patients aged 8–12, and 56.84 ± 24.50 for patients aged 13–18 (Figure 4B). Significant differences were found in total and scale scores among different age range groups, all *p* < 0.05 (Table 6).

**Table 6:** PedsQL GCS total and scale scores by age groups.

Dimension	Physical functioning	Emotional functioning	Social functioning	School functioning	Psychosocial health summary score	Total score
All study population	71.64 ± 22.13	66.66 ± 21.10	69.33 ± 23.83	64.99 ± 21.82	66.83 ± 19.52	68.47 ± 19.42
*n*	187	187	187	178^*^	178^*^	178^*^
PedsQL 2–4 years	76.74 ± 20.31	74.00 ± 20.71	77.22 ± 20.07	79.40 ± 24.60	77.35 ± 18.87	77.38 ± 19.13
*n*	45	45	45	36^*^	36^*^	36^*^
PedsQL 5–7 years	66.00 ± 21.19	64.48 ± 19.07	66.21 ± 24.32	61.72 ± 16.92	64.14 ± 17.93	64.79 ± 18.22
*n*	58	58	58	58	58	58
PedsQL 8–12 years	79.28 ± 17.10	68.68 ± 19.28	70.61 ± 21.38	65.44 ± 16.26	68.25 ± 15.09	72.08 ± 14.26
*n*	57	57	57	57	57	57
PedsQL 13–18 years	59.14 ± 28.03	54.81 ± 24.51	60.19 ± 29.63	51.85 ± 27.18	55.62 ± 24.81	56.84 ± 24.50
*n*	27	27	27	27	27	27
Kruskal-Wallis (*P*)	0.0003	0.0033	0.0254	0.0001	0.0003	0.0003

NF1, neurofibromatosis type 1.

* In PedsQL GCS for patients aged 2–4 years old, school functioning questions were optional and answered only when patients went to school. Nine respondents did not answer school functioning questions.

#### 3.3.3 PedsQL NFM scores for adult patients

PedsQL NFM total score was 68.40 ± 15.57. Among the 18 dimensions of PedsQL NFM, extremely low scores were observed in “worry,” “perceived physical appearance,” and “communication” dimensions (31.63 ± 26.33, 33.67 ± 31.09, 47.03 ± 31.49 respectively) (Table 7). There were significant differences between females and males in total scores, 66.45 ± 15.41 for females and 71.83 ± 15.35 for males (*p* = 0.011). Among the 18 dimensions of PedsQL NFM, significant differences in dimension scores between genders were observed in “skin itch bother” (*p* = 0.014), “pain” (*p* = 0.004), “cognitive functioning” (*p* = 0.010), “perceived physical appearance” (*p* = 0.006), “worry” (*p* = 0.010), “treatment” (*p* = 0.025), “stomach discomfort” (*p* = 0.017) and “constipation” (*p* = 0.002).

**Table 7:** PedsQL NFM total score and dimension scores in NF1 adult patients (*n* = 226).

PedsQL NFM	All adults	Female	Male	*p* value
	*n* = 226	*n* = 144	*n* = 82	
Total score	68.40 ± 15.57	66.45 ± 15.41	71.83 ± 15.35	0.0109^*^
Skin itch bother	67.22 ± 21.66	64.53 ± 21.04	71.95 ± 22.04	0.0139^*^
Skin sensations	74.82 ± 24.08	73.09 ± 23.52	77.85 ± 24.89	0.0765
Pain	74.83 ± 19.39	72.28 ± 19.11	79.32 ± 19.18	0.0038^*^
Pain impact	80.73 ± 20.13	79.71 ± 19.68	82.51 ± 20.90	0.1791
Pain management	79.31 ± 23.35	78.21 ± 22.46	81.25 ± 24.86	0.1760
Cognitive functioning	70.20 ± 20.73	67.51 ± 20.73	74.92 ± 20.00	0.0100^*^
Speech	78.32 ± 20.70	77.82 ± 19.94	79.19 ± 22.07	0.3486
Fine motor	92.61 ± 13.93	93.95 ± 11.55	90.24 ± 17.16	0.1450
Balance	83.98 ± 22.81	85.38 ± 20.90	81.52 ± 25.79	0.4792
Vision	72.46 ± 27.50	70.53 ± 28.02	75.87 ± 26.41	0.1088
Perceived physical appearance	33.67 ± 31.09	29.17 ± 28.72	41.57 ± 33.63	0.0060^*^
Communication	47.03 ± 31.49	44.44 ± 31.02	51.58 ± 31.98	0.1100
Worry	31.63 ± 26.33	28.16 ± 25.25	37.71 ± 27.25	0.0098^*^
Treatment	56.04 ± 28.45	52.80 ± 28.23	61.71 ± 28.11	0.0248^*^
Medicines	83.37 ± 20.28	81.77 ± 21.14	86.18 ± 18.48	0.1492
Stomach discomfort	71.50 ± 24.53	68.81 ± 23.67	76.22 ± 25.43	0.0165^*^
Constipation	74.67 ± 23.61	70.66 ± 25.32	81.71 ± 18.40	0.0018^*^
Diarrhea	79.15 ± 21.12	77.52 ± 21.45	82.01 ± 20.33	0.1354

NF1, neurofibromatosis type 1.

* The ranksum *p* value was < 0.05, significant differences existed between genders.

### 3.4 Caregiver burden: ZBI scores

The average ZBI total score of all caregivers of pediatric patients was 35.26 ± 16.41 (Figure 4C). Total scores of ZBI were 39.47 ± 15.81 for patients aged 0–2, 33.04 ± 14.98 for patients aged 2–4, 37.60 ± 16.26 for patients aged 5–7, 31.84 ± 16.80 for patients aged 8–12, and 35.48 ± 17.98 for patients aged 13–18 (*p* > 0.05).

**Figure 5:**
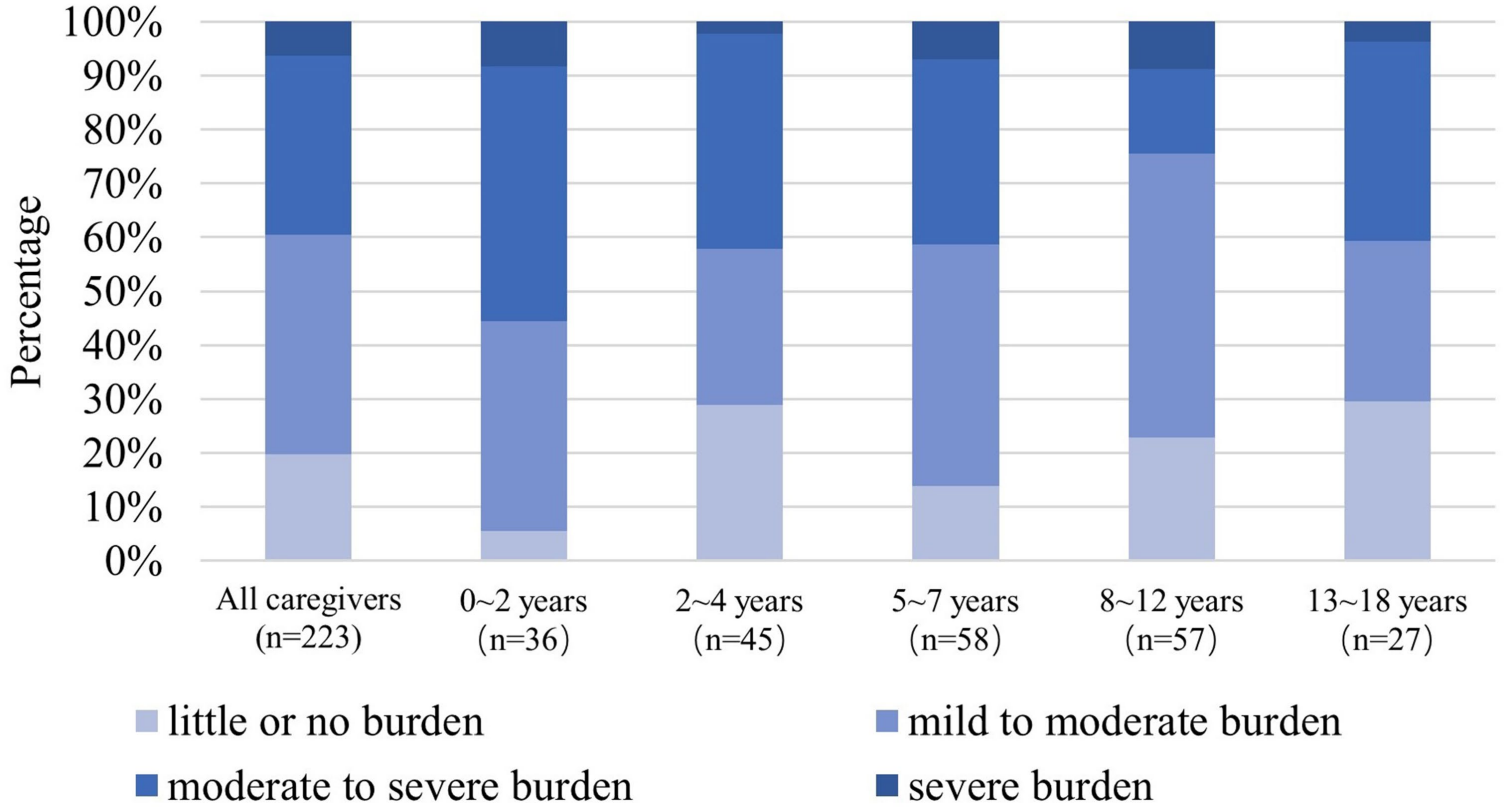
The 100% stacked bar charts show the distributions at caregiver burden levels by age groups according to the ZBI total score (*n* = 223). NF1, neurofibromatosis type 1; ZBI, Zarit Burden Interview.

ZBI scores showed that 39.5% of caregivers had moderate-to-severe or severe caregiver burden (Figure 5). Total ZBI scores reported by mothers and fathers were 36.34 ± 16.17 and 29.95 ± 17.04 (*p* < 0.05), respectively.

## 4 Discussion

This study assessed the disease burden of NF1 patients using economic burden, HRQL, and caregiver burden in a nationwide sample that was relatively large and representative in China. For pediatric patients, the proxy-reported HRQL of children with NF1 and caregiver burden of their caregivers were estimated; for adults, self-reported HRQL was estimated.

Treatments for NF1 vary according to the different clinical manifestations, leading to big differences in individual expenses. Surgery is the primary cost driver of patients’ economic burden in China because it is the main treatment method for NF1 (56). However, the appearance of innovative drugs may change the landscape of NF1 treatments, and thus the main cost driver may change accordingly. More than 60% of NF1 patients had an experience of medical treatment away from home, also called off-site medical treatment, implying unsatisfactory accessibility of treatment for NF1 in most places in China. Since the study was conducted during the period of epidemic prevention and control of COVID-19, off-site medical treatment for NF1 patients was limited, therefore medical expenses could be underestimated compared with the actual demand.

As for HRQL, children with NF1 had a lower EQ-5D-Y utility and EQ-5D VAS score than those of children with hematological malignancies in China (37) but a higher EQ-5D-Y utility than of children with another rare disease, spinal muscular atrophy, in China (57). As for NF1 adult patients, EQ-5D-5L utility was 0.843 ± 0.17 and EQ-5D VAS was 72.32 ± 23.49 in the present study, lower than the EQ-5D-5L norms of China, 0.912–0.971 and 82.9–88.3, respectively (34).

According to the *post hoc* analysis of the present study, NF1 pediatric patients in China had a lower HRQL compared to the published healthy controls although PedsQL total and scale scores were not matched for age and gender. Mean values of total scores and scale scores of NF1 pediatric patients are significantly lower than those of children as healthy controls (all *p* < 0.001) (55). The effect size ranges from −0.54 to −0.83 (Supplementary Table 1). PedsQL scores of the healthy controls in the present study were extracted from the existing published literature which had the largest sample size and most similar age range to ours. PedsQL GCS total scores of healthy children in China ranged from 80.74 to 92.16, all higher than that of NF1 pediatric patients in the present study (55, 58–60). NF1 patients (aged 5–25 years) in the US had a PedsQL total score of 63.47 (61), which is quite close to the result of NF1 pediatric patients in our study.

For pediatric patients, both EQ-5D-Y utility and VAS scores and PedsQL GCS scores hit a trough at the age group of 5–7 years and reached the bottom at the age group of 13–18 years, especially in schooling and emotional functioning. One possible reason for the change of HRQL with age could be the deterioration of NF1 (62, 63). In addition, children go to school initially at the age of 5–7 and step into adolescence and middle school at the age of around 13 in China. In these specific time frames, NF1 pediatric patients, especially those with visible symptoms such as café-au-lait skin spots and neurofibromas that affect their appearance, may suffer from social and emotional distress in school (64). Similar patterns were observed in previous studies, including a study assessing HRQL of children with rare diseases in China using PedsQL GCS (65), and a study assessing HRQL of Finnish children and adolescents using Revidierter KINDer Lebensqualitätsfragebogen (KINDL-R) (66). However, different from our study, a study conducted among NF1 patients aged 5–25 in the US, using self-reported PedsQL GCS, reported that PedsQL GCS total scores of patients with different age ranges were 68.79 ± 19.06 (age 5–7, *n* = 78), 65.86 ± 19.86 (age 8–12, *n* = 97), 66.58 ± 23.30 (age 13–17, *n* = 64), 60.73 ± 21.34 (age 18–25, *n* = 66) (67). The different patterns for HRQL along with age in pediatric patients may related to the various clinical manifestations and different cultural backgrounds. In the upcoming study, it is significant to incorporate diverse stakeholders, notably healthcare providers and educators involved with NF1 pediatric patients, throughout the entire process – from questionnaire design to study implementation to interpretation of the findings.

No significant differences in HRQL were observed between females and males among NF1 pediatric patients, which is consistent with previously published studies that measured the quality of life of children with NF1 (68). However, in adult patients, females were observed to have a lower PedsQL NFM total score than males while no differences between males and females were observed in EQ-5D-5L. Similar results were also observed in a study in Canada (17). In adult populations with different health statuses, females were reported to have lower HRQL than males (69–73).

Caregiver burden in this study, measured as ZBI total score, was higher than that of caregivers of neurofibromatosis type 1 with plexiform neurofibromas (NF1-PN) patients in the US (23.0 ± 13.8) (19). The percentage of caregivers suffering from moderate to severe burden or severe burden in the present study was far higher than the caregivers for children with NF1-PN (19). In our study, patients with or without NF1-PN were included irrespective of the treatments they received. In the study in the US, only caregivers for those pediatric patients who were treatment-naive or received treatment of innovative drugs were included. Whether the use of innovative drugs relieves caregiver burden of NF1 awaits further evidence. Unlike adults, children require continuous physical and emotional care from their parents. As caregivers of pediatric patients play a crucial role, we assessed their burden in the present study. Considering the feasibility, we did not include caregivers of adult patients. However, further research will be necessary to explore the burden on caregivers of adult patients, especially those with severe health conditions.

The present study estimated the disease burden of NF1 patients in China using a relatively large sample size. The HRQL outcomes of this study can serve as a useful reference for populations sharing similar culture with China, especially those facing difficulties in acquiring substantial sample sizes for rare diseases. However, since the healthcare systems vary considerably across countries, applicability should be considered when referring to this study’s direct cost results. Regarding indirect cost, we did not directly assess indirect cost as monetary but reported it as work-loss days and school-loss days, which can reflect the severity of the disease and informal care demand from NF1 patients. These results may be useful for other countries.

The present study had some limitations. In the present study, the HRQL tools for pediatric patients were proxy-reported versions. The proxy version of EQ-5D-Y showed agreement with the self-reported version in published studies (74–76). Although no significant differences in results were found between the proxy version and self-reported version of PedsQL GCS in many studies (55, 77–81), it was generally accepted that proxy version HRQL measures should be used as a supplement of self-reported ones when assessing HRQL of children because inconsistencies between the two versions of HRQL measures were shown in some studies (82–84). According to recommendations, the minimum age for self report of EQ-5D-Y or PedsQL GCS is 8 years. We finally employed the proxy version, after an in-depth discussion with the patient network, for a lot of parents of NF1 patients would conceal the diagnosis from their children out of concern that knowing the diagnosis may affect the children’s psychological health. In addition, there was no existing Chinese version of neurofibromatosis-specific measures for pediatric patients. Therefore, we can only measure their HRQL using generic measures, which may not be sensitive enough to capture NF1’s impact on HRQL.

As for sampling, the population of the present study was recruited from a patient network through mostly online contact. Patients with extremely severe clinical burden or from outlying poverty-stricken areas precluded access to the internet may not be covered in the present study. The included study population covered adult patients who were able to complete the questionnaire and volunteered to participate in the study. Patients with rather severe clinical manifestations and unable to fill out the questionnaire were not recruited in the present study. Therefore, selection bias may exist in the present study and the disease burden may be underestimated. The study did not use a direct measure to assess the severity of NF1 patients since the survey was patient-reported while severity assessment tools of NF1 patients are clinician-reported and patients may not be clear about the nerve and bone symptoms if they did not get a regular check-up near the day of the survey (85).

## 5 Conclusion

The present study estimated the disease burden of NF1 patients using a relatively large and representative sample in China. Results of the study showed that NF1 patients suffered long-term distress throughout their life. The economic burden of NF1 patients, both pediatric and adult, varied widely due to the diverse range of clinical manifestations. The HRQL results were consistent between pediatric and adult NF1 patients, with both groups experiencing low HRQL, particularly in psychological dimension. Caregivers for NF1 pediatric patients had a considerable caregiver burden. More attention and support for NF1 patients are required.

## Data Availability

The original contributions presented in the study are included in the article/Supplementary material, further inquiries can be directed to the corresponding authors.

## Footnotes

^1^https://euroqol.org/register/obtain-eq-5d/

^2^https://eprovide.mapi-trust.org/

^3^http://www.reillyassociates.net/WPAI_Translations.html
