# Supplementary material for: Long-term distress throughout one’s life: health-related quality of life, economic and caregiver burden of patients with neurofibromatosis type 1 in China
Source: Front Public Health. 2024 Aug 21;12:1398803. doi: 10.3389/fpubh.2024.1398803 (PMC11371622; doi:10.3389/fpubh.2024.1398803)
Supplement: Supplementary file 1 [file Data_Sheet_1.docx]

Supplementary Materials

# Supplementary Figures

Figure 1. Histogram of NF1-related overall inpatient cost in 2021 (CNY). Only patients with actual expenses were calculated. *NF1* neurofibromatosis type 1

Figure 2. Histogram of NF1-related overall outpatient cost in 2021 (CNY). Only patients with actual expenses were calculated. *NF1* neurofibromatosis type 1

Figure 3. Histogram of NF1-related formal care cost in 2021 (CNY). Only patients with actual expenses were calculated. *NF1* neurofibromatosis type 1

Figure 4. Histogram of NF1-related other direct medical costs in 2021 (CNY). Only patients with actual expenses were calculated. *NF1* neurofibromatosis type 1

Figure 5. Histogram of NF1-related direct non-medical costs in 2021 (CNY). Only patients with actual expenses were calculated. *NF1* neurofibromatosis type 1

# Supplementary Tables

| **Table 1. Comparing means of total scores and scales scores of PedsQL GCS between NF1 pediatric patients and healthy controls** | | | | | | | |
| --- | --- | --- | --- | --- | --- | --- | --- |
| PedsQL GCS | Neurofibromatosis type 1 | Healthy controls | *t* value | *p* value | Effect size(*d*) | 95% *CI* |  |
| Total score | 68.47±19.42^*^ | 80.74±16.9 | -7.17 | <0.001 | -0.69 | -0.88 to -0.49 |  |
| Physical health summary score | 71.64±22.13 | 82.18±17.6 | -5.73 | <0.001 | -0.54 | -0.73 to -0.35 |  |
| Psychosocial health summary score | 66.83±19.52^*^ | 80.09±17.6 | -7.55 | <0.001 | -0.72 | -0.91 to -0.53 |  |
| Emotional functioning score | 66.66±21.10 | 76.90±16.6 | -5.87 | <0.001 | -0.55 | -0.74 to -0.36 |  |
| Social functioning score | 69.33±23.83 | 85.33±15.4 | -8.85 | <0.001 | -0.83 | -1.03 to -0.64 |  |
| School functioning score | 64.99±21.82^*^ | 78.03±16.5 | -7.28 | <0.001 | -0.70 | -0.89 to -0.50 |  |

*NF1* neurofibromatosis type 1; PedsQL GCS PedsQL^TM^ 4.0 Generic Core Scales

The sample size for Neurofibromatosis type 1 was 187 and the sample size for the health controls was 284.

*Nine respondents did not answer school functioning questions so the sample size for these scale scores was 178.
