# Supplementary figures and images for: Long-term distress throughout one’s life: health-related quality of life, economic and caregiver burden of patients with neurofibromatosis type 1 in China
Source: Front Public Health. 2024 Aug 21;12:1398803. doi: 10.3389/fpubh.2024.1398803 (PMC11371622; doi:10.3389/fpubh.2024.1398803)

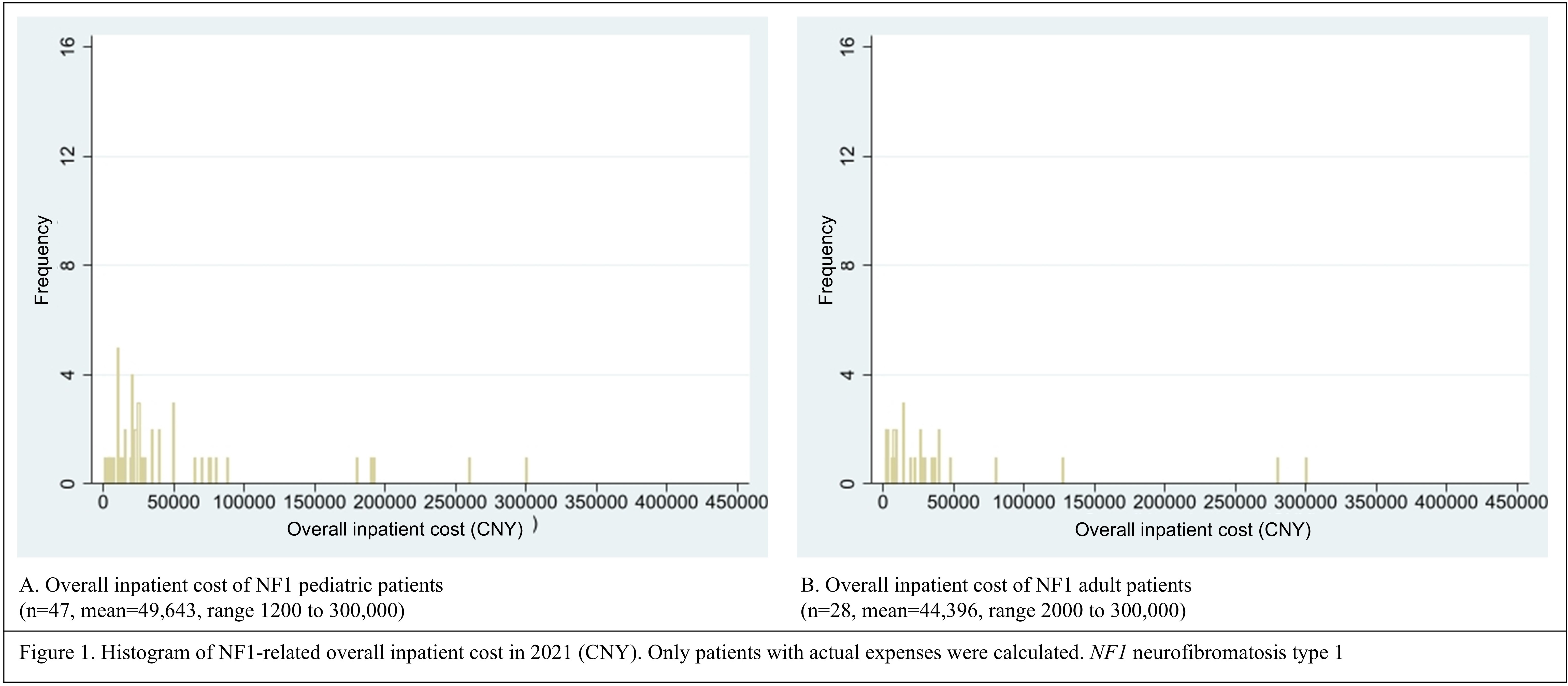

Supplement: Supplementary file 2 [file Image_1.jpg]

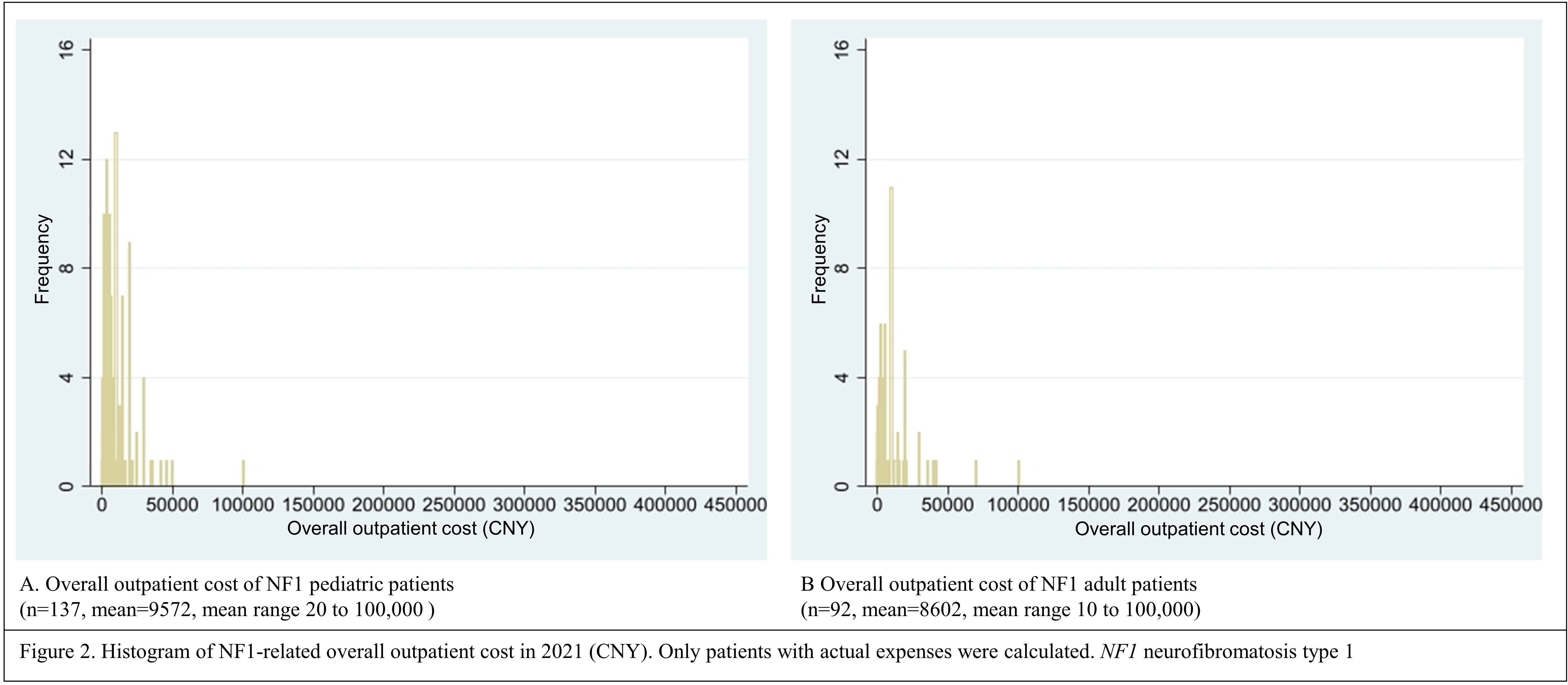

Supplement: Supplementary file 3 [file Image_2.JPEG]

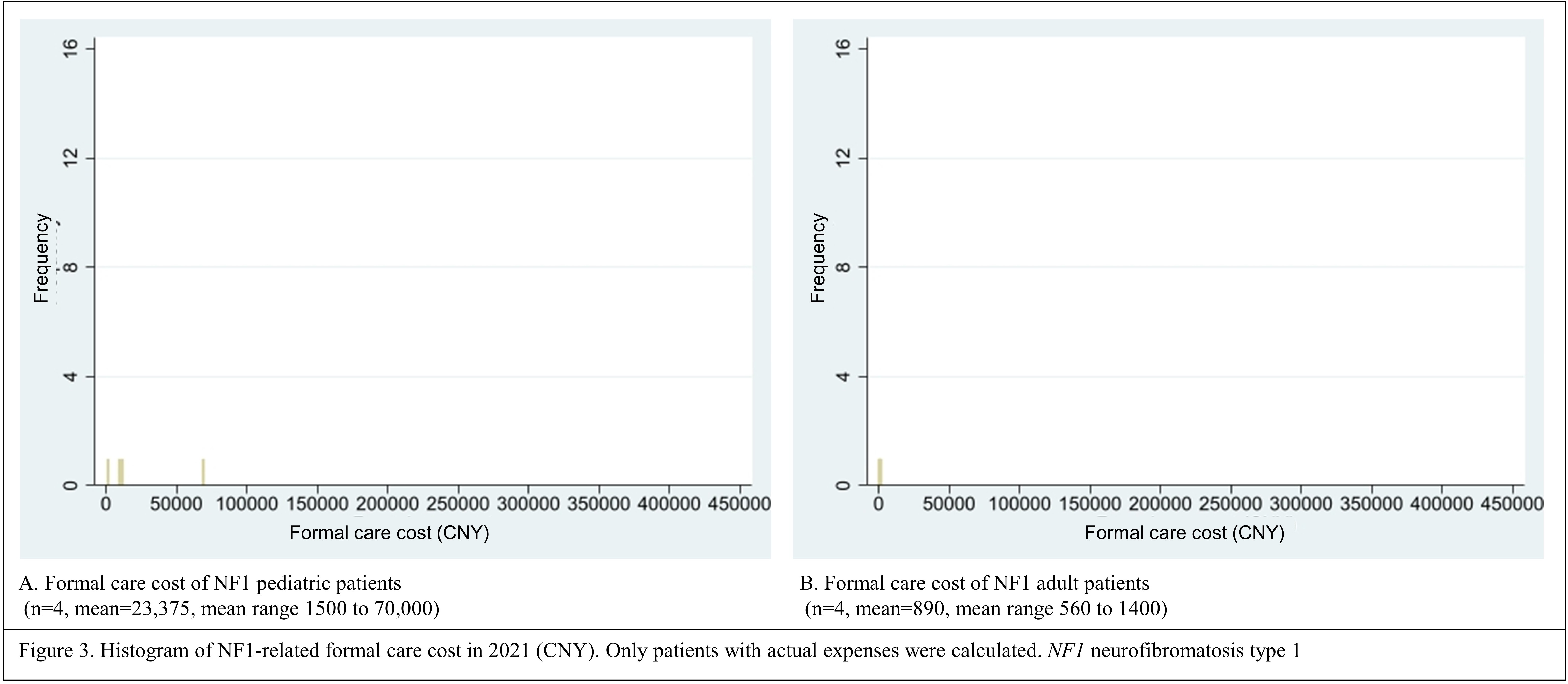

Supplement: Supplementary file 4 [file Image_3.JPEG]

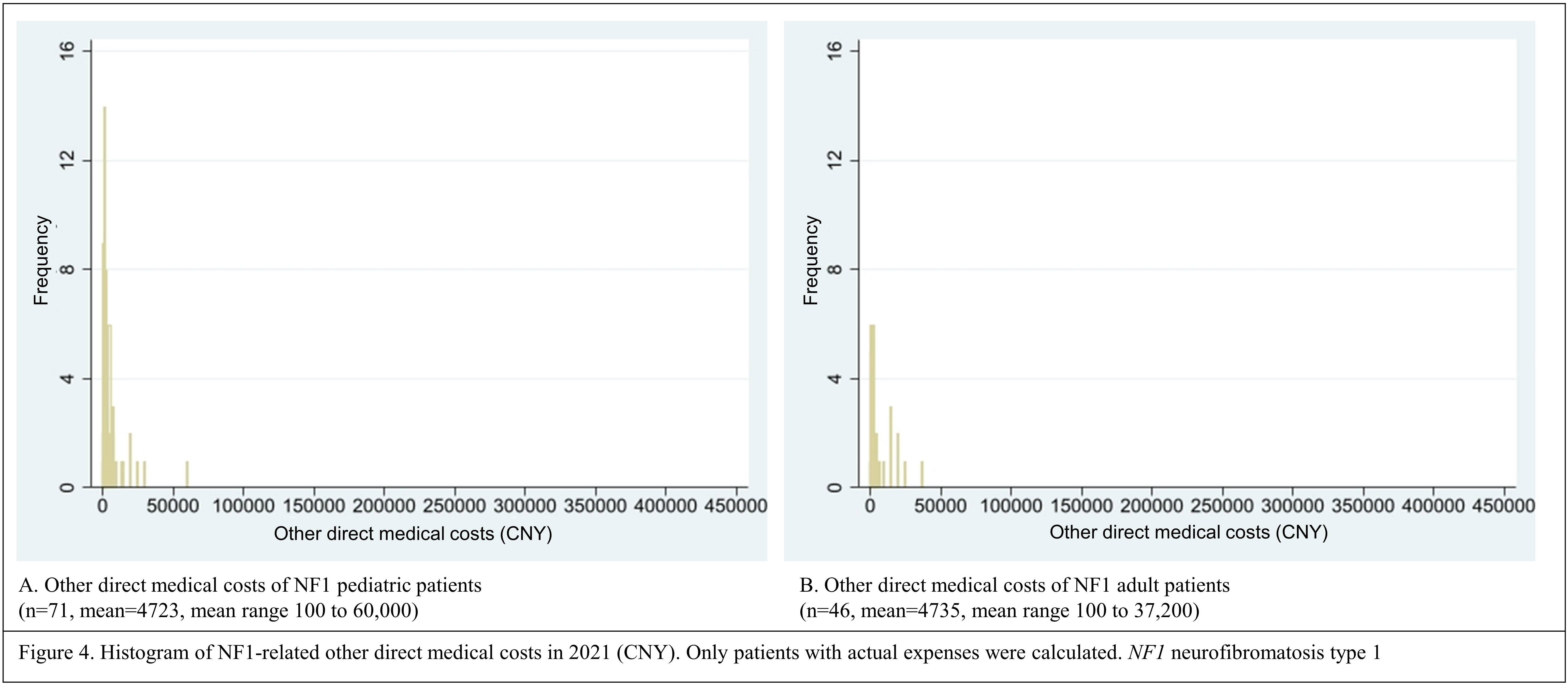

Supplement: Supplementary file 5 [file Image_4.JPEG]

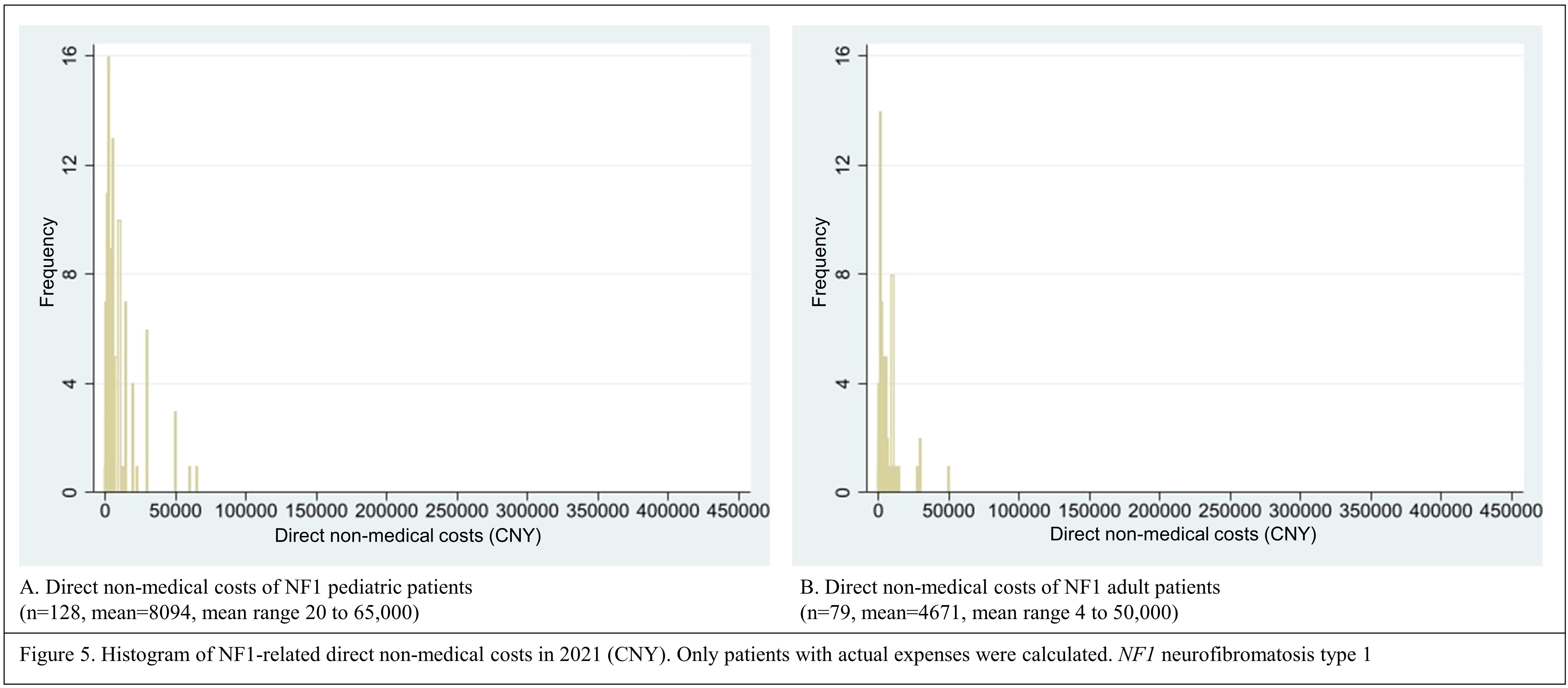

Supplement: Supplementary file 6 [file Image_5.JPEG]
